# Supplementary material for: Identification of Novel Genes and Proteoforms in Angiostrongylus costaricensis through a Proteogenomic Approach
Source: Pathogens. 2022 Oct 31;11(11):1273. doi: 10.3390/pathogens11111273 (PMC9694666; doi:10.3390/pathogens11111273)
Supplement: Supplementary file 1 [file pathogens-11-01273-s001.zip › Supplementary.pdf]

Supplementary Information

Identification of novel genes and proteoforms in Angiostrongylus costaricensis through a proteogenomic approach

Esdras Matheus Gomes da Silva, Karina Mastropasqua Rebello, Young-Jun Choi, Vitor Gregorio, Alexandre Rossi Paschoal, Makedonka Mitreva, James H. McKerrow, **Ana Gisele da Costa Neves-Ferreira, Fabio Passetti**

This file contains the description of:

- **Supplementary Figures S1-6**
- **Supplementary Tables S1-2**
- **Supplementary Data S1-13**

Supplementary Figures

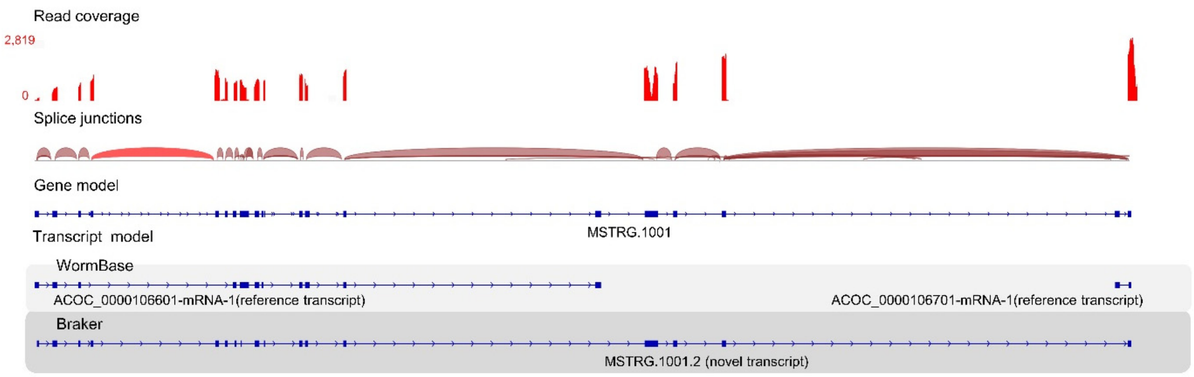

Supplementary Figure S1. Example of a WormBase split gene merged by BRAKER annotation.

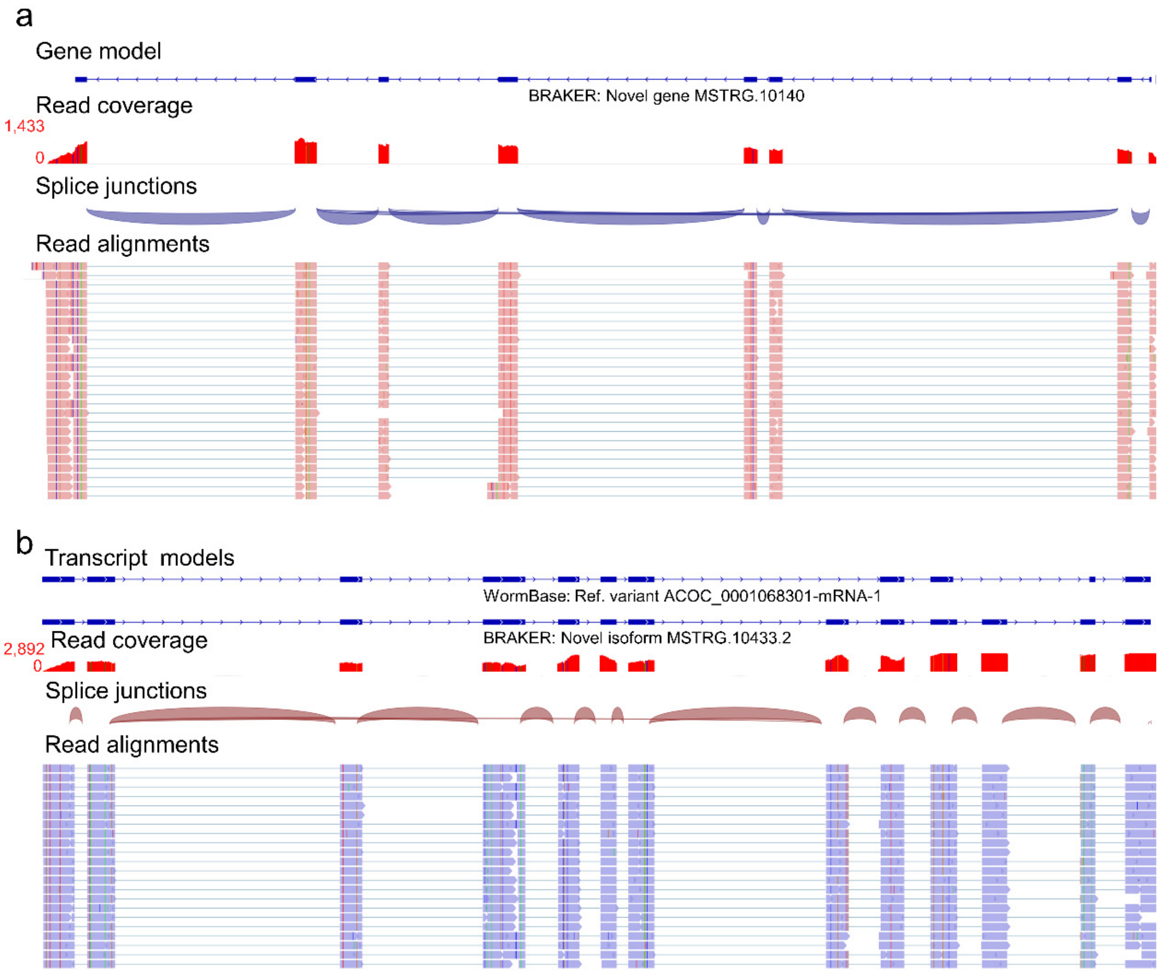

Supplementary Figure S2. Visual representation of gene/transcript models and RNA-Seq alignments. **a)** Novel gene predicted by BRAKER with the support of RNA-Seq reads. **b)** Novel transcript variant predicted by BRAKER with the support of RNA-Seq reads.

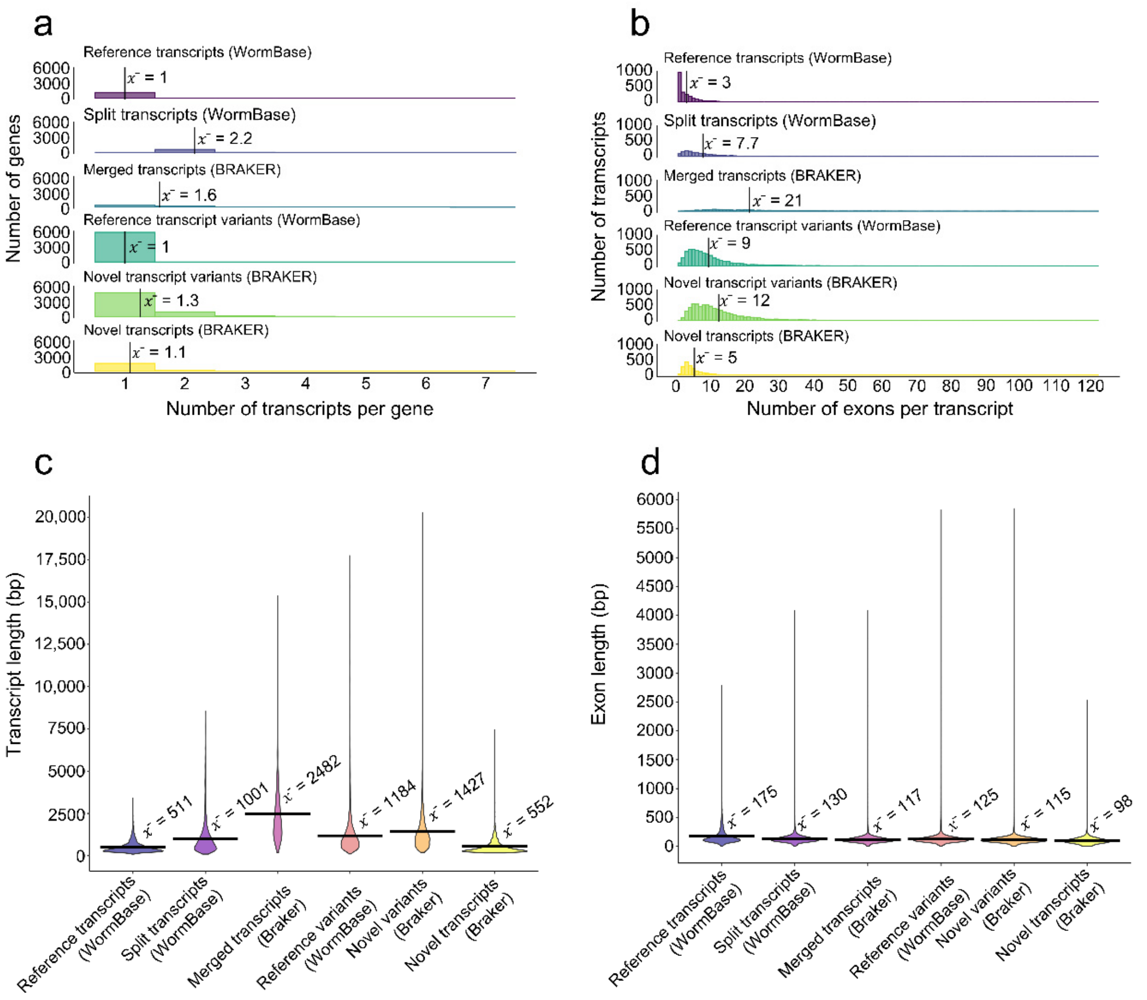

Supplementary Figure S3. Distribution of the different features of the supplemented genome annotation**. a)** The histogram represents the distribution of the number of transcripts per gene. **b)** The histogram represents the distribution of the number of exons per transcript. **c)** The violin plot represents the transcript length distribution measured in base pairs (bp). **d)** The violin plot represents the exon length measured in base pairs (bp). Categories: Reference transcripts (WormBase), transcripts exclusive to the WormBase prediction; Transcripts from split genes (WormBase), WormBase transcripts that overlap BRAKER transcripts; Merged transcripts (BRAKER), BRAKER transcripts that overlap WormBase transcripts; Reference transcript variants (WormBase), transcript variants exclusive to the WormBase annotation; Novel transcript variants (BRAKER), transcript variants exclusive to the

BRAKER annotation; Novel transcripts (BRAKER), transcripts exclusive to the BRAKER annotation.

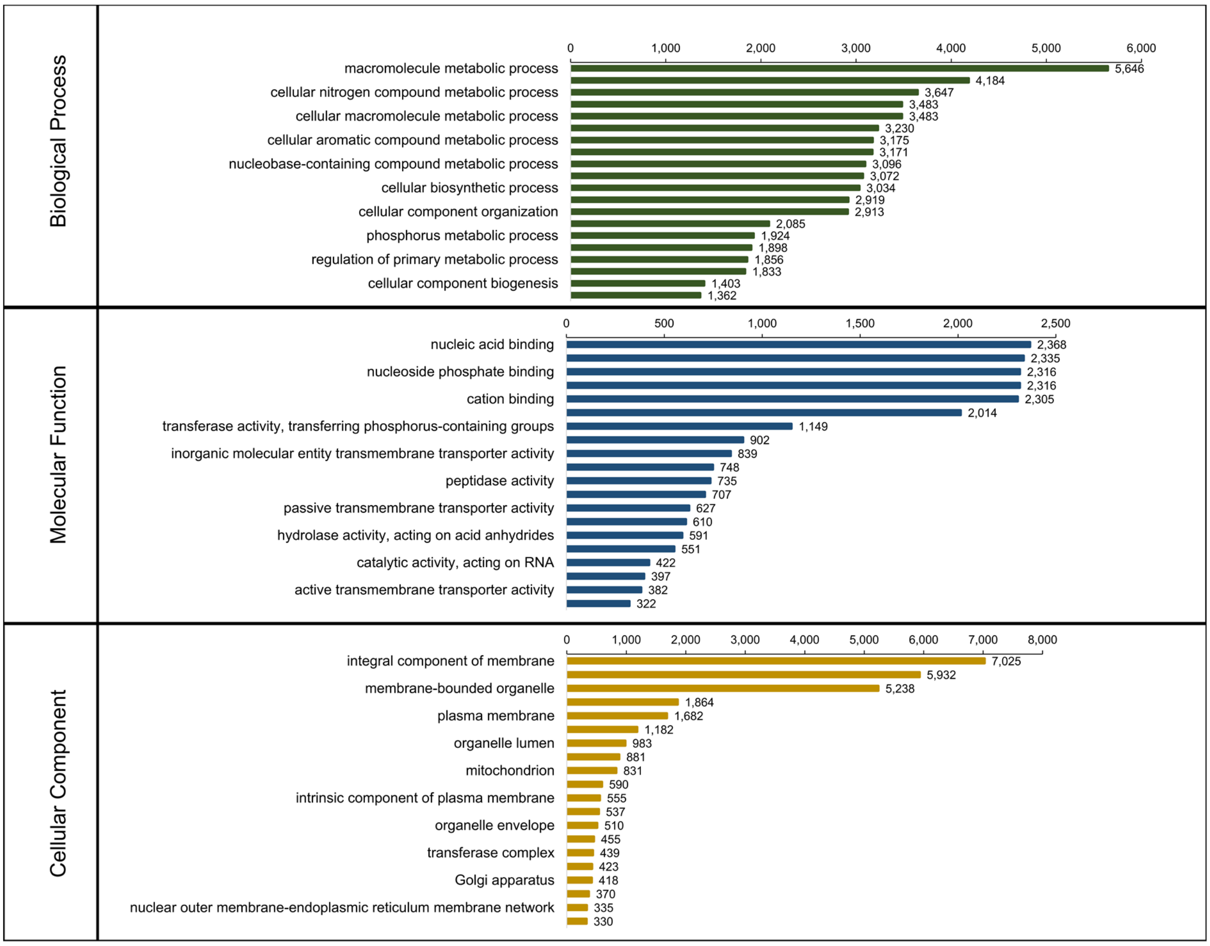

Supplementary Figure S4. Distribution of top 10 Gene Ontology (GO) terms. The 4th level of GO terms is presented as Biological Process, Molecular Function, and Cellular Component.

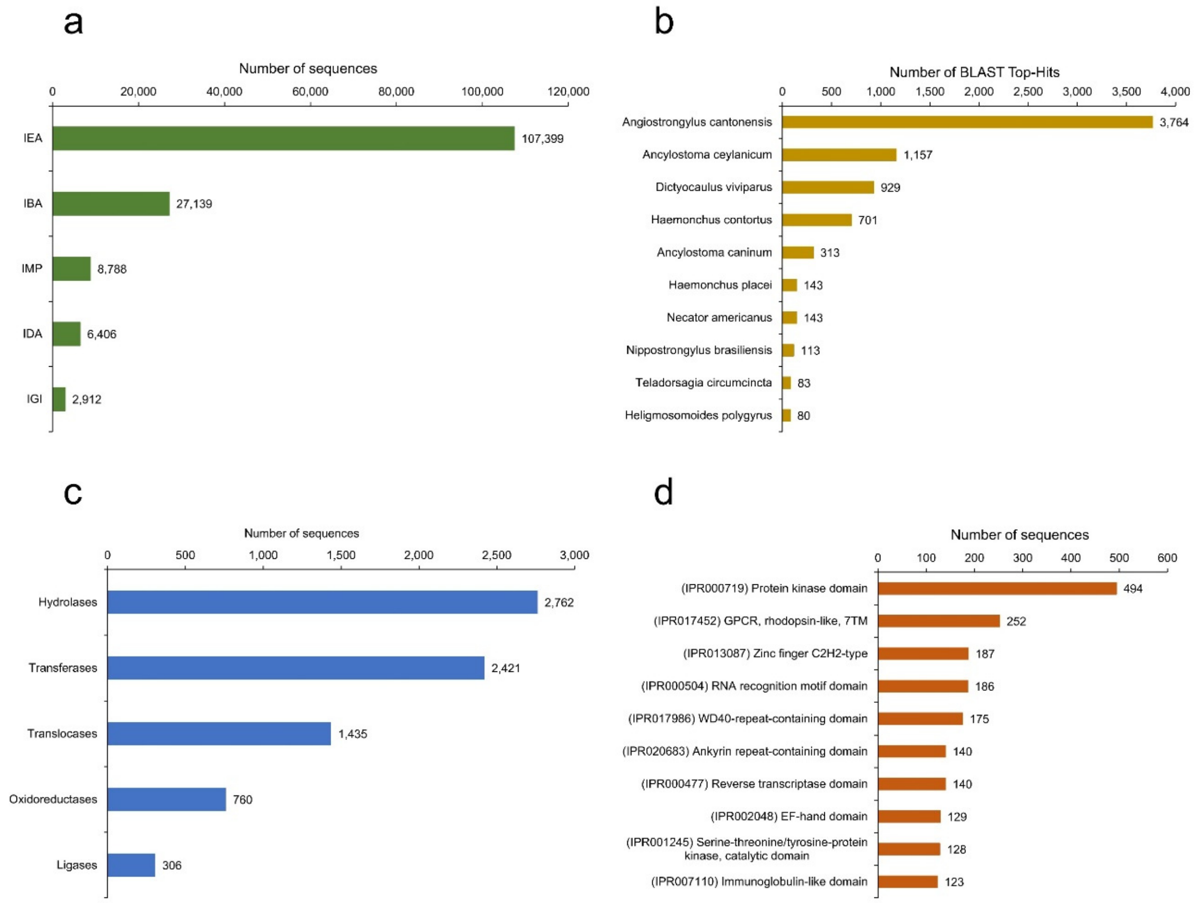

Supplementary Figure S5. Blast2GO annotation. **a)** Top 10 number of protein sequences by annotation evidence code: Inferred from Electronic Annotation (IEA), Inferred from Biological aspect of Ancestor (IBA), Inferred from Mutant Phenotype (IMP), Inferred from Direct Assay (IDA), Inferred from Genetic Interaction (IGI). **b)** Top 10 number of BLAST top-hits by species. **c)** Top 5 number of protein sequences by enzyme annotation. **d)** Top 5 number of protein sequences by InterProt domain annotation.

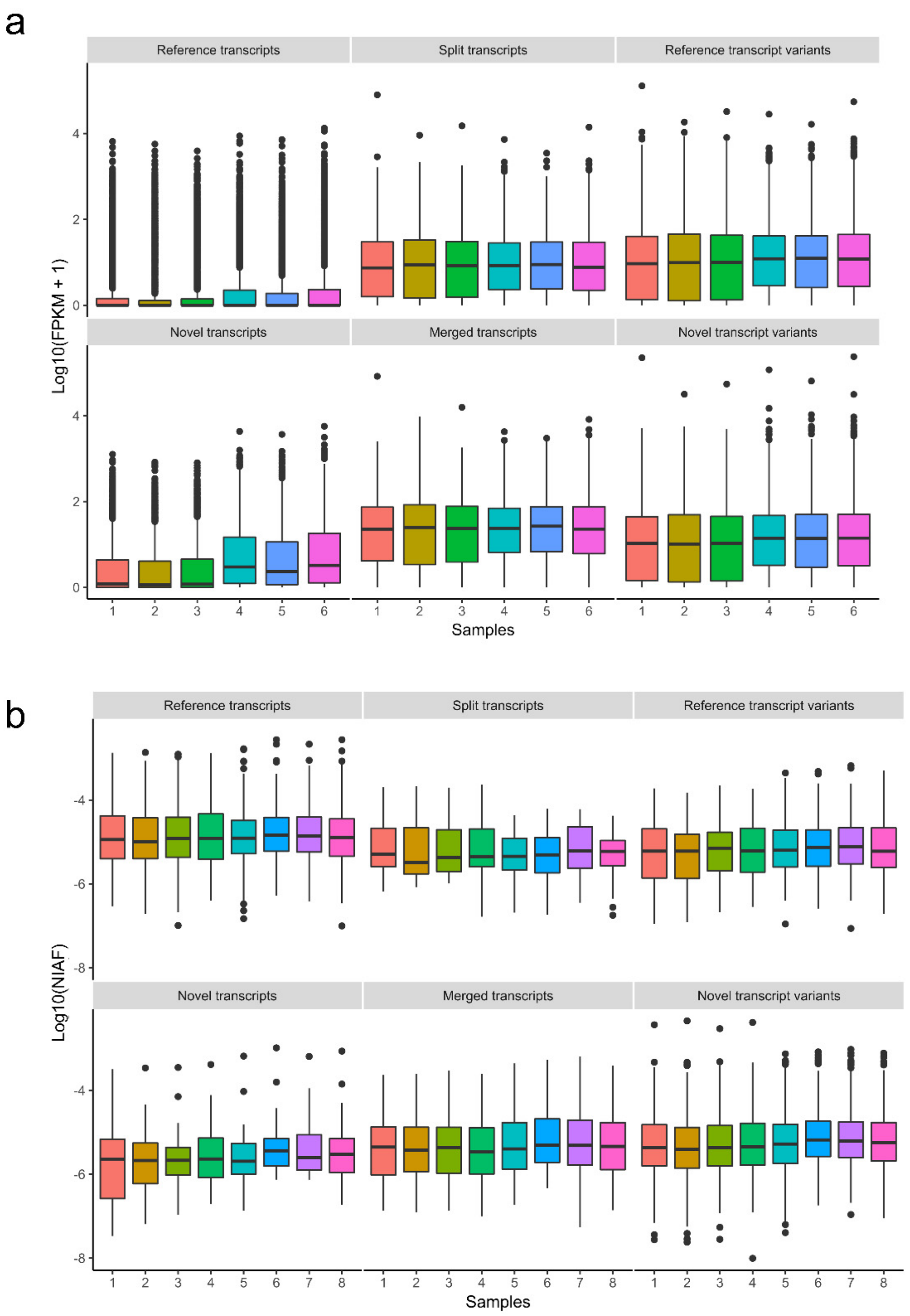

Supplementary Figure S6. mRNA and protein abundance levels following *A. costaricensis* transcriptomic and proteomic analyses. **a)** Box plot of mRNA normalized abundance level (FPKM) of each biological replicate. **b)** Box plot of protein normalized abundance level (NIAF) of each biological replicate. mRNA and proteins were categorized as follows: Reference transcripts (WormBase), transcripts exclusive to

WormBase prediction; Transcripts from split genes (WormBase), WormBase transcripts that overlap BRAKER transcript; Merged transcripts (BRAKER), BRAKER transcript that overlaps WormBase transcripts; Reference transcript variants (WormBase), transcript variants exclusive to WormBase annotation; Novel transcript variants (BRAKER), transcript variants exclusive to BRAKER annotation; Novel transcripts (BRAKER), transcripts exclusive to BRAKER annotation.

Supplementary Tables

Supplementary Table S1. Distribution of RNA-Seq reads mapping onto *A. costaricensis* WormBase reference genome sequence and assigned to a gene.

Samples	Trimmed reads	Uniquely mapped reads	Reads assigned to a gene
		Count (rate)	Count (rate)
1	41,913,390	31,619,730 (75%)	22,393,341 (53%)
2	35,201,977	29,245,656 (83%)	13,312,178 (38%)
3	44,645,242	32,317,621 (72%)	25,845,175 (58%)
4	39,211,262	24,708,796 (63%)	20,774,227 (53%)
5	48,852,398	34,514,824 (71%)	29,800,454 (61%)
6	42,949,668	25,839,339 (60%)	21,609,207 (50%)

Supplementary Table S2. Comparison of the number of ncRNA genes from *A. costaricensis*, *C. elegans* and *H. contortus*. *A. costaricensis* [FPKM] represent ncRNA genes with evidence of expression.

ncRNAs	*A. costaricensis*	*A. costaricensis* [FPKM]	*C. elegans* (Ensembl)	*H. contortus* (WormBase)
antisense_RNA	0	0	104	0
Cis-reg	4	2	0	0
lincRNA	0	0	184	0
miRNA	8	2	718	0
ncRNA	5	5	7779	0
piRNA	3	3	15363	0
rRNA	19	14	22	2
snoRNA	1	1	346	0
snRNA	8	0	129	0
sRNA	1	0	0	0
tRNA	377	7	634	22

Supplementary Data

Supplementary Data S1. WormBase

Supplementary Data S2. Split genes merged by BRAKER

Supplementary Data S3. Shared genes

Supplementary Data S4. BRAKER

Supplementary Data S5. Transcript lengths and their transcript categories

Supplementary Data S6. Protein identifications using the Wormbase database

Supplementary Data S7. Top 10 Gene Ontology (GO) terms by the 4th level

Supplementary Data S8. Blast2GO functional annotation

Supplementary Data S9. Nucleotide and hypothetical amino acid substitutions per transcript

Supplementary Data S10. Nucleotide and amino acid substitutions per transcript and their identified peptides

Supplementary Data S11. Normalized transcript levels (FPKM) of each quantified transcript

Supplementary Data S12. Normalized protein levels (NIAF) of each quantified protein

Supplementary Data S13. Mean FPKM and NIAF of each cluster
